# ERRATUM: Avaliação de três métodos de extração de DNA de *Salmonella* sp. em ovos de galinhas contaminados artificialmente

**DOI:** 10.29374/2527-2179.bjvm001625

**Published:** 2025-03-14

Due to the absence of ACKNOWLEDGMENTS in the article “Avaliação de três métodos de extração de DNA de *Salmonella* sp. em ovos de galinhas contaminados artificialmente” (https://bjvm.org.br/BJVM/article/view/370), published in Brazilian Journal of Veterinary Medicine, v. 37, n. 2, p. 115-119, 2015, please, consider the following:

AGRADECIMENTOS

Os autores agradecem o apoio financeiro concedido pela Fundação de Amparo à Pesquisa do Estado de Minas Gerais (FAPEMIG, Belo Horizonte, MG, Brasil), Conselho Nacional de Desenvolvimento Científico e Tecnológico (CNPq, Brasília, DF, Brasil) e Coordenação de Aperfeiçoamento de Pessoal de Nível Superior (CAPES, Brasília, DF, Brasil).

The authors apologize for the errors.

